# Factors associated with increasing rural doctor supply in Asia-Pacific LMICs: a scoping review

**DOI:** 10.1186/s12960-020-00533-4

**Published:** 2020-12-01

**Authors:** Likke Prawidya Putri, Belinda Gabrielle O’Sullivan, Deborah Jane Russell, Rebecca Kippen

**Affiliations:** 1 Department of Health Policy and Management, Faculty of Medicine, Public Health and Nursing, Universitas Gadjah Mada, IKM Building 2nd Floor, Jl. Farmako, Sekip Utara, 55281 Yogyakarta, Indonesia; 2 School of Rural Health, Monash University, 26 Mercy Street, Bendigo, VIC 3550 Australia; 3 Rural Clinical School, Faculty of Medicine, The University of Queensland, Toowoomba, Australia; 4 Menzies School of Health Research, Darwin, Australia

**Keywords:** Physicians, Rural health services, Professional practice location, Career choice, Developing countries

## Abstract

**Background:**

More than 60% of the world’s rural population live in the Asia-Pacific region. Of these, more than 90% reside in low- and middle-income countries (LMICs). Asia-Pacific LMICs rural populations are more impoverished and have poorer access to medical care, placing them at greater risk of poor health outcomes. Understanding factors associated with doctors working in rural areas is imperative in identifying effective strategies to improve rural medical workforce supply in Asia-Pacific LMICs.

**Method:**

We performed a scoping review of peer-reviewed and grey literature from Asia-Pacific LMICs (1999 to 2019), searching major online databases and web-based resources. The literature was synthesized based on the World Health Organization Global Policy Recommendation categories for increasing access to rural health workers.

**Result:**

Seventy-one articles from 12 LMICs were included. Most were about educational factors (82%), followed by personal and professional support (57%), financial incentives (45%), regulatory (20%), and health systems (13%). Rural background showed strong association with both rural preference and actual work in most studies. There was a paucity in literature on the effect of rural pathway in medical education such as rural-oriented curricula, rural clerkships and internship; however, when combined with other educational and regulatory interventions, they were effective. An additional area, atop of the WHO categories was identified, relating to health system factors, such as governance, health service organization and financing. Studies generally were of low quality—frequently overlooking potential confounding variables, such as respondents’ demographic characteristics and career stage—and 39% did not clearly define ‘rural’.

**Conclusion:**

This review is consistent with, and extends, most of the existing evidence on effective strategies to recruit and retain rural doctors while specifically informing the range of evidence within the Asia-Pacific LMIC context. Evidence, though confined to 12 countries, is drawn from 20 years’ research about a wide range of factors that can be targeted to strengthen strategies to increase rural medical workforce supply in Asia-Pacific LMICs. Multi-faceted approaches were evident, including selecting more students into medical school with a rural background, increasing public-funded universities, in combination with rural-focused education and rural scholarships, workplace and rural living support and ensuring an appropriately financed rural health system. The review identifies the need for more studies in a broader range of Asia-Pacific countries, which expand on all strategy areas, define rural clearly, use multivariate analyses, and test how various strategies relate to doctor’s career stages.

## Introduction

Continuing to strengthen the rural health workforce is crucial as part of building universal health coverage and achieving Sustainable Development Goals [1–4]. Even when rural people, who account for nearly half of the world’s population, are covered by universal health insurance, service coverage may be poor without a sufficient number of skilled local health workers [3, 5].

Higher doctor-to-population ratios correlate with lower maternal, child, and neonatal mortality [6, 7] and lower all-cause morbidity and mortality rates [8, 9], suggesting that access to tertiary qualified doctors is essential. Countries at all levels of socioeconomic development are investing in strategies to improve the supply and retention of qualified doctors in rural areas. High-income countries such as the United States, Canada, and Australia have implemented numerous policies and published extensively about various interventions, including financial incentives, rural education pathways, regulatory, and personal and professional support strategies to address rural doctor shortages [10–15]. In low- and middle-income countries (LMICs), stand-alone policies of compulsory rural healthcare-professional placements have also been implemented [16, 17]. However, the range of research informing how to improve access to qualified rural doctors in LMICs remains to be summarized. An additional quality issue is the lack of evidence relating to doctors at various career stages, since medical workforce dynamics may change by stage professional development [18].

The Asia-Pacific region is home to more than half of the global population, with approximately 98% of the Asia-Pacific population living in 29 LMICs, and just over half of these LMIC populations living rurally [19]. Doctor-to-population ratios in Asia-Pacific LMICs are well below the World Health Organization (WHO)’s benchmark of 1.15-to-1000 population [19], which is essential to achieve its Sustainable Development Goals [1]. Thus, it is critical to understand the effectiveness of strategies implemented to increase rural medical workforce supply in Asia-Pacific LMICs.

With this background in mind, this review summarizes and synthesizes existing evidence about factors associated with preferences and actual work locations of medical students and doctors in Asia-Pacific LMICs. This is done with a view to identifying effective strategies for recruiting and retaining doctors in rural areas. Additional aims are to describe how studies define rural or remote and to determine the spread of evidence by career stage, so as to inform how to target strategies better.

## Methods

### Nature of review

The scoping review method was used as it was most relevant to answering the primary research question about the range and extent of existing evidence. Scoping reviews, unlike traditional systematic reviews, place less emphasis on the critical appraisal of the included evidence, thus allowing the inclusion of a broader range of literature potentially relevant to capturing emerging evidence in Asia-Pacific LMICs [20]. The protocol for this review was developed iteratively by the authorship team according to Preferred Reporting Items for Systematic Reviews and Meta-analysis Extension for Scoping Review (PRISMA-Scr) [21].

### Search strategy

The authors, with assistance of an experienced librarian, developed a Boolean string from key search terms (Table 1) and tested hits against ten key articles known to the first author with 100% sensitivity. Included were terms that covered LMICs, sub-regions and country groups in the Asia-Pacific as at 2019, using the World Bank 2019 definition of Asia-Pacific LMICs [22]. Other search terms addressed the population of interest (medical doctors), exposures of interest, and location of practice.

**Table 1:** Search terms applied in the scoping review

	Key concepts	Related keywords	Related subject term
1	Population	Doctors; general practitioner; medical practitioner; medical graduate; physician; primary care physician; medical officer; medical intern	General practitioners; family physicians; primary care physicians; medical staff; medical graduate; medical practitioner; medical intern; health personnel; health workshop; internship and residency
2	Concept: exposures of interest	Recruit; recruiting; recruitment; retain; retention; turnover; attrition; career; work location; practice location; geographic distribution; geographic imbalance; shortage	Career choice; job satisfaction; motivation; personnel development; choice behavior; physician incentive scheme
3	Concept: location of practice	In rural or remote or non-metropolitan or non-urban or underserved or underserviced or regional	Rural health services; rural health; professional practice location; medically underserved area; rural population
3	Context	Each of the low and middle-income countries in Asia-Pacific region, as defined by the World Bank that include South Asia as well as East Asia and Pacific [22]: Afghanistan, Bangladesh, Bhutan, Cambodia, China, Fiji, India, Indonesia, Kiribati, DPR Korea, Lao, Malaysia, Maldives, Marshall Island, Micronesia, Mongolia, Myanmar, Nauru, Nepal, Pakistan, Palau, Papua New Guinea, Philippines, Samoa, Solomon Island, Sri Lanka, Thailand, Timor-Leste, Tonga, Tuvalu, Vanuatu, Vietnam	Developing countries; low and middle-income countries; Asia; Asia-Pacific; East Asia; South Asia; Southeast Asia; Western Pacific; Pacific Islands; Oceania

Both peer-reviewed and non-peer-reviewed literature published in the last 20 years (July 1999–June 2019) were retrieved. Pubmed, Medline, CINAHL, EMBASE, PsycINFO, Web of Science, and SCOPUS were searched. *Human Resources for Health* and *Rural and Remote Health* journals were also searched. Grey literature searches included Proquest dissertations; first 10 pages of Google Scholar for each country; hrhresourcecenter.org (category: rural/urban imbalance, deployment); WHO website; and Global Health Workforce Alliance website. We searched the eligible articles’ references to identify any additional materials.

### Study selection

Included were studies investigating the following outcomes: (1) actual work, referring to current work, and; (2) preference, referring to attitude towards, intention to work and remain, in the rural and remote areas. As the 2010 WHO global policy recommendations to improve rural health worker recruitment and retention emphasize the importance of educational interventions, and to specifically explore career stage [23], the review included studies of doctors, medical students and interns. This review was restricted to university-qualified doctors or students undertaking tertiary (university-level) degree training (Table 2).

**Table 2:** Inclusion and exclusion criteria applied in the scoping review

	Inclusion criteria	Exclusion criteria
1	English language	Non-English language
2	Year of publication 1999–2019 (July 1999–June 2019)	Year of publication before 1999 or after July 2019
3	Countries of study classified as low- or middle-income (LMICs) and were located in the Asia-Pacific region, as defined by East Asia and Pacific and South Asia region by the World Bank [22]	Countries of study classified as not LMICs, or not East Asia and Pacific or South Asia, or LMICs in Asia-Pacific that are overseas territories of other nations, or include populations from LMICs in Asia-Pacific but not separately reporting the results for this group from other non-LMICs or non-Asia-Pacific countries
4	Investigates outcome as preference or intention (i.e., attitude towards or intention for rural work, intention to stay) or actual work (i.e., current rural work or rural retention)	No preference or intention for rural work or staying or actually working in rural location reported
5	Investigates work in rural or in any remote area or in any underserved area defined by geographic-related criteria or by the authors or reports for rural, remote or underserved separately	Investigates work in metropolitan or in unspecified geographic location without separately reporting work in rural, remote or underserved locations
6	Exploring factors related to the outcome as mentioned in criteria 4	Did not explore factors related to the outcome, for example: presented the proportion of respondents working in rural location without exploring why
7	Analyzes the results for either doctors who completed their medical qualifications at tertiary (university) level, or medical students training at tertiary level, or both	Articles including other types of health workers without separate reporting for tertiary qualified doctors or medical students
8	Full-text of the articles available using online database, browsing or accessible through the University	Full-text irretrievable after attempting searching online database, browsing or via support from the University library
9	Empirical research or review article with a clear search strategy	Non-empirical or review articles without clear search strategies

After retrieving articles and removing the duplicates, we screened titles and abstracts against inclusion/exclusion criteria. We worked independently, then in pairs, to compare assessments and reach agreement. When eligibility for inclusion differed, the team conferred to resolve difference.

### Data charting and analysis methods

A spreadsheet of key factors was developed to extract relevant data. Two authors tested and refined the data extraction tool using 10 eligible studies. Information extracted covered key areas such as country, sample, rural work outcomes, and factors related to the outcomes, and organized using the categories and sub-categories of the WHO global policy recommendations [23].

To understand how eligible articles defined ‘rural’ or ‘remote’ areas, we searched for explicit and implied definitions in the text according to categories described in previous studies such as: non-metropolitan area, population density and characteristics, distance from the nearest town and environmental characteristics [24, 25].

The authors discussed, agreed on, summarized and synthesized findings and implications for future research, policy and practice. Although not the main purpose of this scoping review, an overall exploration of study quality was undertaken to identify issues in research quality and support ongoing research.

## Results

### Source of evidence

**Fig. 1:**
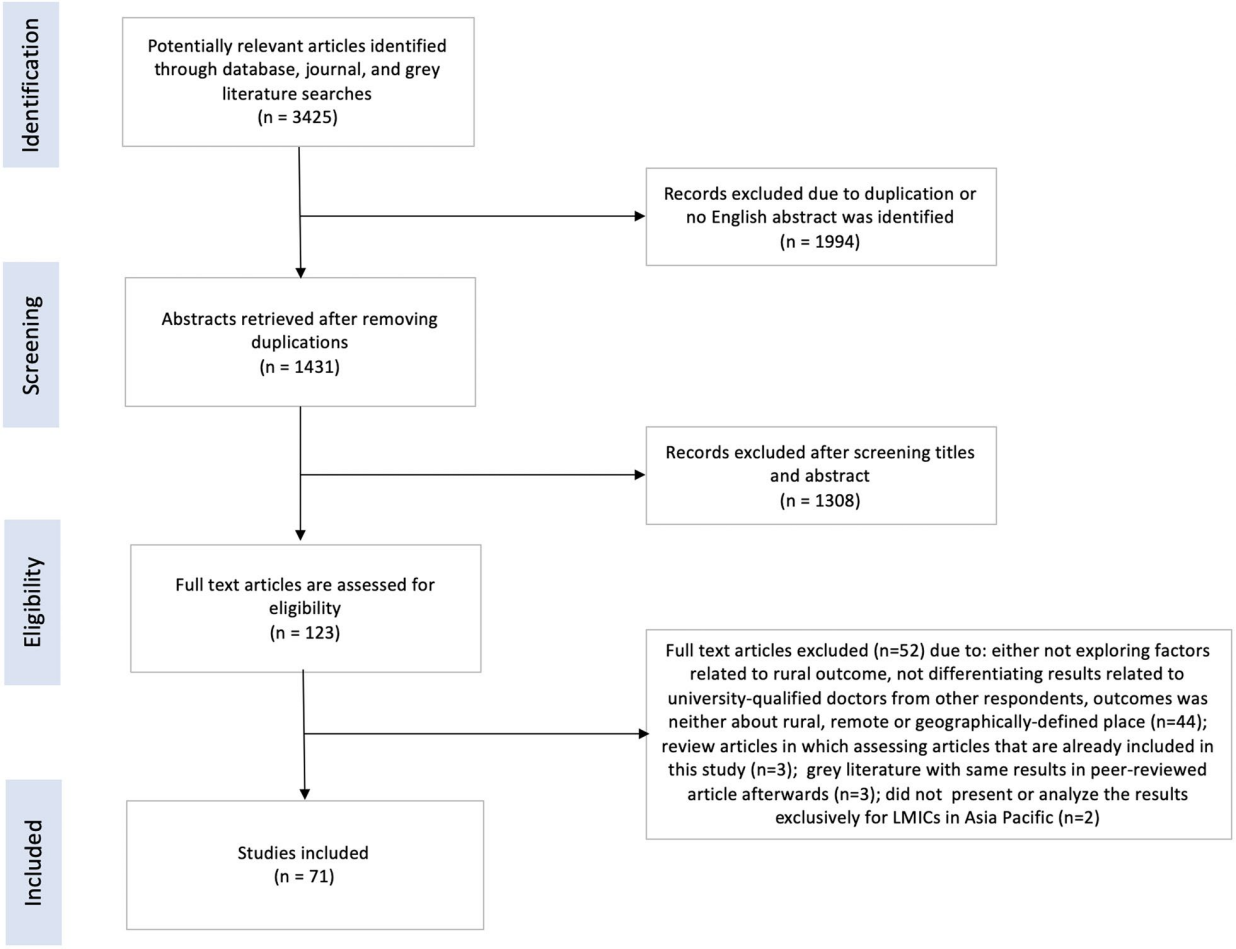
PRISMA diagram of article selection process

**Fig. 2:**
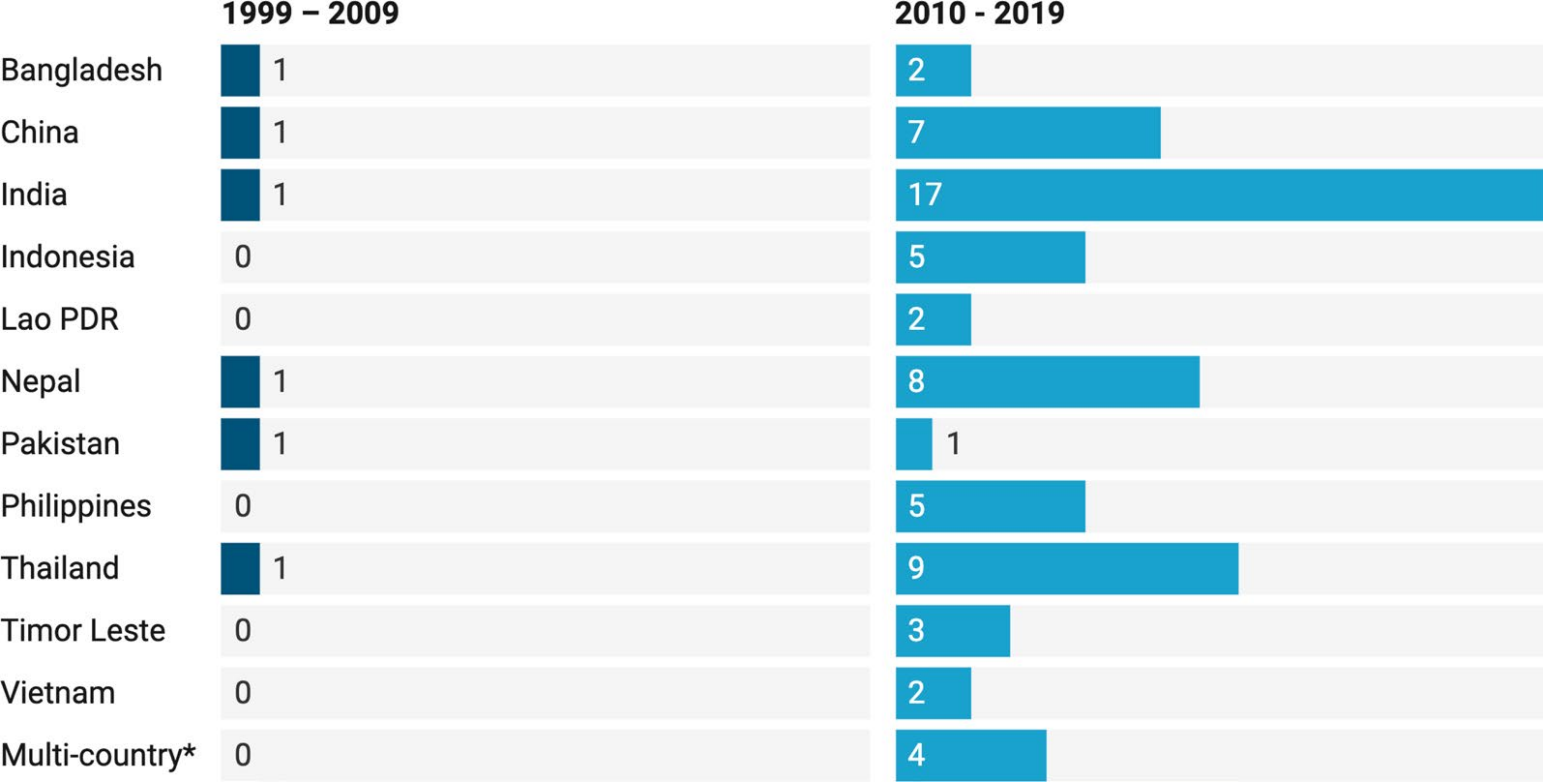
Peer- and non-peer-reviewed articles on effective rural medical workforce strategies by country and year. *Countries included in multi-country studies: Bangladesh (3 articles), Cambodia (1 article), China (2 articles), India (3 articles), Nepal (2 articles), Thailand (2 articles), and Vietnam (2 articles)

The search retrieved 3425 articles. After removing duplicates and screening titles and abstracts, 71 articles were included in this review (Fig. 1). Ninety-two percent of the 71 eligible articles (see Additional file 1) were published after 2009 (Fig. 2).

Although the search was for low- and middle-income country studies, Nepal was the only low-income country (LIC; as classified in 2019) addressed in articles that met the search criteria. However, Nepal has been classified as a middle-income country (MIC) since 2020. No study from Pacific Island nations met the inclusion criteria.

### Study characteristics

The majority (69%) of studies were quantitative, about one-quarter (24%) were qualitative, with the remainder (7%) policy analyses and review. More than half of studies (62%) included only tertiary-qualified doctors (at any career stage) as respondents, one-quarter (25%) of studies included only medical students and nine studies included both. Of 53 studies involving doctors (44 studies with doctors only and 9 studies with both doctors and students), half (53%) explored preference for rural work while the remaining (47%) investigated actual rural work (Table 3).

**Table 3:** Summary of the characteristics of the eligible articles

Data collection method and analysis		Population analyzed				
		Medical graduates^a^		Medical students^b^	Both medical graduates and students	
Method	Number of studies	Preference^c^	Actual^d^	Preference^c^	Preference^c^	Actual^d^
Quantitative	48	12	19	16	5	0
Descriptive analysis	12	3	9	1	0	0
Univariate analysis only	10	3	4	1	3	0
Multivariate analysis	26	6	6	14	2	0
Qualitative	15	4	6	2	3	2
Policy analysis	3	1	2	0	0	0
Review	2	0	2	0	0	0
Mixed method	3	1	2	0	0	0
Total	71	44		18	9	

71 articles were originated from 12 countries: 1 low-income country (Nepal), and 11 middle-income countries (Bangladesh, Cambodia, China, India, Indonesia, Lao, Pakistan, Philippines, Thailand, Timor-Leste, and Vietnam)

^a^Includes: doctors at internship; any doctors before, during and after attending postgraduate study or specialization

^b^Includes: medical students at all levels

^c^Preference refers to intentions or attitude to work or stay working in rural or remote locations among respondents who were not working in such locations at the time of data collection

^d^Actual refers to current work or retention in rural or remote locations at the time of data collection

### Factors associated with doctors working in rural locations

We present results of factors associated with preference or actual work in rural locations according to the WHO areas (educational, regulatory, financial incentives, and professional and personal support), and an additional category of factors related to health system contexts. Some rural work predictors identified in the MIC-based studies—such as rurally located medical school, rural clerkship and rural internship—were not identified in the studies of Nepal (the only LIC in studies that met the search criteria). Differences were due to the scope of covariates explored. Given this, and because only one LIC was included in the studies considered, we present the results of both low- and middle-income countries as a unified analysis.

There were mostly concurrences in factors associated with preference and actual rural work when studies of similar factors were explored as to their findings. Therefore, we discuss the findings in an integrated way while noting them separately in Table 4. Where relevant, any differences in preference and actual rural work outcomes are discussed.

**Table 4:** Factors associated with actual/preferred work in rural locations of Asia-Pacific LMICs medical graduates and students

Category based on WHO framework [23]	Summary of findings	Actual^a^	Preference^b^
A. Education			
1. Factors related to selecting students with particular characteristics			
a) Students with rural backgrounds†	Rural background was defined as either born, spent most of childhood, or finished high school in rural locations	Overall, only 1 study, in Nepal, had proven association of rural background, as a stand-alone factor, and rural practice [26]. The rest studies [27–32] presented strong association between being enrolled under the special track, comprising rural recruitment, scholarship and receiving a rurally enhanced curricula, and rural work	There are mixed findings about association between rural recruitment and rural work preference. While the majority of studies agree that having rural background is associated with rural work preference [33–46], three studies found no difference [47–49]
b) Students who are native to specific locations‡	Native is defined as respondents’ places of origin, where they lived during childhood or prior entering medical school, without mentioning the rurality of the location	Three studies revealed that one of the reasons to decide work location was because they are native to that particular area [50–53]	Studies showed association between being native to and intention to work rural areas [48, 49], or preference for rural practice increased if posted in or near to native area [54], however, another found the opposite [55] Rao et al. revealed while respondents are 1.2 – 2 times more likely to prefer rural work when it is located in their native area, but no difference in this association was found between those with and without rural upbringing [56]
c) Students with certain parental socioeconomic or educational backgrounds‡	Parental socioeconomic or educational background refers to level of income, educational attainment and sector of income	No study had explored parental socioeconomic and educational backgrounds	Students who prefer rural work were more likely to: have a parent with a lower educational background [38, 40, 46]; have parents who are farmers [37]; have parents with low or medium wealth [43, 44], and; do not have a relative who is a doctor [43]. However, some studies found no association between rural work and: parental income level [33, 34, 37, 39]; educational background [35, 48, 49]; parent’s job as a civil servant [49]; and having a parent who is physician [55]
d) Family location‡	Location of family, including parents, spouse, children, or extended family. The different findings may be due to differing definitions of first-degree and extended family members used	Three qualitative studies revealed that location of family have been among the most frequent reasons of doctors chose working rurally [51, 52, 57]. Family location was also associated with working in rural locations [43, 45]	Doctors whose family members are in urban areas regard urban jobs more highly than those without family in urban areas [58]. Likewise, medical students’ decisions about work location were associated with the location of family [36, 40, 42]. It was also uncovered that location of family as one of main reasons of intention to work in rural locations [49, 59] Some studies, however, found no association between having extended family in rural locations and intention to work rurally [33, 34]
e) Type of entry to medical school ‡	Some countries allow different types of entry to medical school, such as direct (5 years of medical course for those completing high school) or non-direct or graduate entry (3 years of medical course for those with some tertiary degree). Some schools also offered regular and international program, in which the international program had a higher tuition fee	No difference of rural practice between doctors with paramedical or science tracks [26]	Better preference of rural work was found among those: attending the graduate entry compared to direct entry [47]; enrolled under the regular program compared to those in international program [42]
f) Other aspects that were found as strong predictors: type of high school, personal characteristics, specialty	Type of high school refers to the public (government) or private ownership of the school. Specialty was type of specialization pursued after completing a medical degree	No studies had explored association between actual rural work and type of high school Two studies unveiled that doctors working in rural locations because of sense of altruism and spiritualism [50, 51, 60]	Medical students graduating from government secondary schools are more likely to have rural work intentions [43] Doctors or students with personal characteristics of altruism, optimism, higher self-efficacy, or self-decision-making were more likely to be willing to work in rural areas [34, 37, 47, 53, 59, 60] Doctors on a GP track compared prefer rural work more compared to those in clinical medicine and public health tracks [38]
g) Other aspects with no association ‡		A higher academic performance during medical school was not associated with actual rural work [26]	A higher academic performance during medical school was not associated with rural work preference [43]
2. Factors related to delivering educational programs			
a) Health professional schools outside of major cities †	Location of medical school considered as ‘outside of major cities’ were: outside of capital, or any rural or remote locations as defined by the studies	Doctors graduated from medical school outside the capital cities were more likely to work rurally [46, 61]. Other studies from Thailand and the Philippines found that establishing of medical schools in rural locations, combined with rurally enhanced curricula, were associated with increased doctor supply in rural locations [27–31, 62–65]	One study found that those studying in medical schools in rural locations were more likely to prefer rural work [40], while no difference on rural work preference between newly graduates from rural and urban medical school [48, 49]
b) Clinical placements (clerkships) in rural areas during studies †	Clerkship is a clinical placement or rotation phase, usually took place in the final year(s) of study. During the clerkship, students were rotating in different departments and treating patients under supervision	Being enrolled in a special track, comprising rural recruitment, rural medical school, rural clerkship, scholarship tie to compulsory service [19–22], and rurally enhanced curricula [62–65], was associated with rural work. No study had explored how a rural clerkship, as a stand-alone factor, was associated with actual rural work	Rural clinical clerkships were found to be associated with rural preference for students with an urban background [38]. Being enrolled in special track, comprising rural recruitment, rural medical school, rural clerkship, scholarship tie to compulsory service was associated with rural preference [25, 26]
c) Curricula that reflect rural health issues †	Curricula designed for rural- or community-based comprising: additional or extended exposures to community or rural settings	Curricula designed for rural- or community-based medical education—whether combined with other educational interventions such as scholarships [66], spending part of medical school in rural locations [62–65], or as a stand-alone intervention [30]—was associated with better doctor supply in rural areas	A fellowship program in a rural hospital, contains a community-based project work, exposures to cases in a rural (secondary hospital), has improved positive attitude toward rural career [67]
d) Continuous professional development for rural health workers †	Professional development refers to activities to improve skills and knowledge of health workers including short-term and long-term trainings, postgraduate study and specialization	Doctors in rural locations are less likely to have opportunities for postgraduate training compared to those in urban locations [68]. Evidence indicates that guaranteed professional development (i.e., continuing education, a higher score for postgraduate enrollment) was related to doctors’ rural work [50, 60, 66, 69–71] or staying in rural locations [53, 72]	Opportunities for professional development, whether of short duration like workshops or longer duration like postgraduate study, is one of the pivotal attributes considered by doctors in deciding to work [54, 56, 58, 60, 73, 74] or stay working [16, 50] in rural or remote locations Medical students are more likely to prefer rural posts if being offered opportunities to continue education or enhance their professional development [47, 59, 75–79]
e) Rural internship‡	Medical internship is a phase medical graduates have an official medical doctor degree (such as MBBS or MD) but have yet to obtain license to practice unsupervised	Rural internships, delivered with rurally enhanced curriculum, were found to be positively associated with subsequent rural work [64]	Unpleasant experience while completing internship program in rural areas had discouraged doctors to continue working there [80]
f) Students from certain type of medical school ‡	Type of medical school refers to the public or private ownership of the school	No study had explored association between actual rural work and type of medical school	Two studies showed that students in public medical schools are more likely to prefer rural work compared to those in private schools [43, 55], whereas another study found no difference [56]
B. Regulatory			
a) Compulsory service †	Compulsory service refers to any posting mandated for doctors with full practice license	Compulsory rural service policies post-graduation for 1–3 years have a positive association with increased rural doctors in Thailand [31, 81, 82]	It was found that those having completed 2 years of compulsory service were more likely to prefer rural jobs compared to those who completed 1 year [54]
b) Subsidized education for return-of-service †	Return-of-service refers to an obligatory assignment for doctors who received scholarships	Doctors [66, 81] who received a scholarship tied to compulsory service were more likely to stay working in rural compared to their counterparts Being enrolled in a special track, in which the scholarship tied to compulsory service provided for those recruited from rural areas, was associated with improved rural doctor supply [27–30, 72]	Students [43, 47] who received a scholarship tied to compulsory service were more likely to prefer rural work compared to those without scholarship Being enrolled in a special track, in which the scholarship tied to compulsory service provided for those recruited from rural areas, in addition to being recruited from rural areas and received a rurally enhanced curriculum, was associated with better rural preference [48, 49]
C. Financial incentives			
a) Appropriate financial incentives †	Financial incentives refer to salary, hardship allowances or any additional money received by doctors with regard to their service in rural locations	Rural doctors, despite longer total working hours, received less income compared to the urban doctors [46]. The financial incentives (i.e., salary, hardship allowances) were among major attributes or challenges of doctors working in rural or remote locations [50, 52, 53, 70, 83]. The increasing incentive with remoteness was associated with an increase of rural doctor supply in China [32, 84]. However, financial incentives were deemed less valuable for retention compared to other factors such as working environment, community and personal factor among rural doctors in Thailand [72]	Appropriate financial incentives (i.e., salary, hardship allowances) were associated with doctors preference to work [36, 46, 84] or staying in rural locations [45, 85]. Better financial incentives were desired by doctors [52, 54, 56, 58, 60, 73, 74, 85–87] and medical students to address rural doctor shortages [36, 40, 47, 59, 75–80, 88, 89]
b) Opportunity to earn additional income ‡	Opportunity to additional income refers to income-generating activities related to clinical service, usually in private sector, hence the term ‘private practice’	Government doctors working in rural areas have a more limited opportunity for private practice [32, 50, 73, 90]. While a study in Pakistan revealed that private practice was one of reasons of willingness to work in rural areas in Pakistan [45], a study in India discovered that aversion to private practice was among reasons of doctors chose to work in rural location [53]	Lacking private practice opportunity in rural areas has discouraged interns to continue working in rural locations [80]
D. Personal and professional support			
a) Better living conditions †	Better living conditions refers to any environmental aspects related to personal amenity such as housing, transportation, electricity, water and communication, education and business facility	Any general aspects of poor living conditions [45, 50, 52, 57, 72, 83], schooling facilities [50, 53], spouse employment [53], access to electricity and water supply [36], transportation [49], were among the key reasons for unwillingness to work rurally	There is evidence that preference to work in rural locations is associated with: short travel time to work [91], availability of transportation for official and unofficial use [76], positive perception of living conditions [47], and good educational facilities and connectivity [56]. However, in other studies, associations were not found between rural preference and: housing allowance or support [58, 75], access to a vehicle [58] and spouse and child education [34] Overall better living conditions [6, 10, 11, 35, 45, 65, 70, 71], housings [76, 92], basic infrastructure (i.e., electricity, water, communications connectivity) [52, 57, 59, 88], transportation [57, 72, 74, 76], access to nearest town [41], and children schooling facilities [73], were also important attributes to rural preference. Females regarded housing provision higher than males [58, 74]
b) Safe and supportive working environment†	Working environment comprising both human and non-human resource such as: other health or non-health professionals, facility infrastructure, drugs and medical equipment	Despite the same average working hours in their main job, doctors in rural areas had longer working hours in dual practice compared to urban doctors [46]. Higher rural doctors’ workloads, owing to inadequate supply of health professionals in rural location or difficult geographical access, was another reason doctors were unwilling to work or remain in rural posts [50, 52, 57, 70, 93] Other important attributes for rural doctor recruitment was lack of drugs, equipment and facility infrastructure [52, 57, 66, 83], while for retention was good relationships with peer and manager [53, 72]	One study found that higher satisfaction score to work environment were associated with intention to stay working in rural area [91] Other attributes important to improve intention to work or staying in rural areas were: adequate number of health professional [73, 85], relationship with colleagues or seniors [80], lack of drugs, equipment and poor facility infrastructure [40, 59, 60, 73, 79, 81, 88, 92, 94] Of those studies applying discrete choice experiment methods, 2 studies found that an adequate health facility was less important to medical students than salary [75, 76], while 2 studies found the opposite among doctors [56, 58]
c) Foster interaction between urban and rural health workers†	Interaction between urban and rural health workers comprising communication or consultation of doctors in rural areas with specialists or others with higher skills in urban areas	Limited access to highly skilled colleagues was among explanations discouraging doctors to work in rural areas [70, 73]	Access to specialists or consultant was mostly considered important for increasing preference to rural work [58, 79], though, it was off less importance when compared to increased salary, posting near home province, opportunity to continue to specialization and career promotion [54]
d) Career ladders†	Career ladder refers to career path that promotes doctor to a higher position, which is generally have better salary and benefit	Poor career ladder schemes were one of reasons hindering doctors to work rurally [50, 52, 57, 70, 83]. One of Thai government’s policy to improve rural recruitment was to provide opportunity for rural doctors to attain a high position, equivalent to that in urban location [31]	Creating a clear career ladder is important to improve doctors’ preferences to work in rural or remote locations [36, 47, 59, 60, 73, 79, 85, 86, 88, 92, 93] The following are examples of career promotion schemes preferred for rural doctor recruitment: associated with higher rural work preferences were: promoted as permanent staff [54, 76], possibility to transfer to other more developed areas after certain period of employment [56]
e) Professional network†	Professional network refers to opportunity to connect and communicate with other peers in rural health service	A study found professional isolation was a deterrent to work rurally [52], while the presence of network of rural doctor is believed to improve doctors’ willingness to practice rurally [31]	A disconnected health services between urban and rural was among the prioritized attributes desirable by doctors for working in rural areas. [88]
f) Public recognition†	It refers to official or non-official recognitions received by the doctors	Rural doctors acknowledged the lack of recognition, especially as a primary care doctor, as one of challenges working in rural locations [57, 83]. An award for rural doctors was one of policies in Thai that was believed increased rural doctor supply [31]. Another Thai study confirmed that professional recognition was among important attributes for doctors to stay in rural region [72]	No study had identified public recognition as the major attribute for rural work preference
g) Security‡	It refers to situations related to personal safety of the doctors	Lack of security has been one of deterrents to work in rural or remote locations [50, 72], and two studies in India highlighted that this issue was especially raised by female respondents [57, 83]	Poor security was among major issues that should be tackled to improve doctors preferring to work in rural areas [40, 73, 79, 88, 92]
h) Community support‡	Community support comprising appreciation, reception, support, literacy, language and cultural compatibility	Connection and the absence of language barrier with community [83], or community appreciation, growth and support [72], was important attributes in attracting doctors to work or remain in rural locations	Community appreciation, literacy, attitude to western medicine have been mentioned as one of factors motivated doctors and students to work in rural locations [39, 41, 79, 89]
i) Career stages‡	Career stages refers to the length of employment as a doctor	No study had explored association between actual rural work and career stages	While one study found doctors completed 2 years compulsory service are more likely to prefer rural jobs compared to those with doctors completed 1 year [54], another one found no difference between career stages [91]
j) Human resource management‡	This refers to the management of hiring, firing and incentivizing health workers, usually performed by local or national government	No study had explored association between actual rural work and local-level human resource management	Although the definition is unclear, support from local government was among the most selected attributes influencing rural work preferences of medical students [75] and intention for rural retention among doctors [80, 85]
k) Gender‡		There is evidence that male are more likely to working in rural areas [27, 29]	Half of the studies investigating gender (*n* = 16) found no difference in rural preference between male and female [33, 34, 36, 39–42, 44–46, 48, 49, 55, 56, 91, 93]. While some studies found that, after adjusting for other variables, being male was strongly associated with preference to work in rural areas [43, 47, 77], several other studies found the opposite [35, 37]. Some DCE studies also found that males and females value job attributes differently: males are more responsive to workplaces near their home province and salary increases [54, 58], while females value housing more highly [58, 74]
l) Marital status‡		None had investigated association between marital status and actual rural work, despite such information was collected in the survey or interview	While one study found a weak association between being unmarried and willingness to take a rural job [45], most found no difference in rural preference or work according to marital status [33, 34, 39, 44, 88, 91]
E. Health systems‡			
a) Governance‡	This refers to any aspect related to leadership and governance beyond the health facility	Political favoritism was shown to interfere with processes and procedures for doctors’ career development in rural locations [50, 70, 83]	It was perceived that political interference and instability were attributable to poor rural work preference [59, 73, 93]
b) Service delivery (organizational policy)‡	This refers to aspects related to health service organization and management	Changes in hospital autonomy, allowing more flexible financial management, has supported hospitals in urban areas to recruit more doctors thus reducing those working in primary healthcare clinics in rural areas [32]. Privatization of rural health have been associated with doctors moving to bigger health facilities in urban locations and away from rural health facilities [95]	No study had investigated the service delivery-related aspects with regard to rural work preference
c) Health financing‡	This refers to any aspect related to financing of the health systems	Capitation of staff at the hospital level had encouraged urban hospitals that were typically overstaffed to cease doctor recruitment, which could have resulted in more doctors working in rural hospitals [31]. In Cambodia, the government developed a package of healthcare services that required doctors to provide care at the hospitals. This policy resulted in doctors moving to hospitals, which were located in urban areas, and thus was associated with reduced availability of doctors in rural areas [32]	No study had investigated the health financing-related aspects with regard to rural work preference

*DCE* discrete choice experiment

^a^Actual refers to current work or retention in rural or remote locations at the time of data collection

^†^The category was included in the WHO Global Policy Recommendation

^‡^Additional category based on the results of the scoping review

### Educational

Eighty-two percent of the articles—from 12 Asia-Pacific LMICs—were about educational factors, categorized into 2 areas: (1) student selection, and; (2) delivering medical education. For student selection, most studies demonstrated rural background was associated with both rural preference [33–46, 54] and actual work [26], while several found no association with rural preference [47–49, 56]. Being enrolled through the 'special track', which consists of rural student recruitment, scholarships and receive a rural-oriented curriculum, were associated with actual work in rural areas [27–32]. Other student selection factors associated with rural preference were: having parents with lower educational level or wealth [38, 40, 43, 46] or with an income source from the agricultural sector [37, 44], entering medical school through a graduate track [47], and having graduated from a government-owned high school [43]; however, there was no evidence for such association with the actual work. Studies exploring the delivery of medical education found associations for both rural preference and actual work with rurally located medical schools [40, 46, 61], rural clerkship [37], and rural-oriented curricula combined with other educational strategies [27–31, 62–65]. Students in public medical schools were more likely to prefer rural work compared to those from private ones [43, 55]. Rural internship was cited as a negative experience leading to poorer intention to work rurally in Indonesia [80], while, when delivered as part of a rural-oriented curricula, it was associated with better rural doctor supply [64].

### Regulatory

One-fifth (20%) of articles, from China, Nepal, Thailand, and Timor-Leste, examined regulatory strategies. Compulsory rural service periods, whether implemented as a stand-alone strategy [31, 81, 82], combined with scholarships only [43, 47, 66, 81], or combined with combined with scholarship and recruiting students from rural areas [27, 29–31, 47–49, 72], were associated with higher rural preference or actual work.

### Financial incentives

Forty-five percent of the studies—from 12 Asia-Pacific LMICs—explored associations between rural preference or actual work and financial incentives. Despite many demonstrating that appropriate financial incentives were essential for rural doctor recruitment, it was not clear what increment of incentive was needed for optimal results. One study revealed the actual income is higher among urban than rural doctors [46]. Across studies applying discrete choice experiment (DCE) methods, the proportion of increased salary or allowances tested varied, ranging from 0 to 300%. One study demonstrated doctors and medical students were 1.1–1.3 times more likely to consider rural jobs if offered 16% higher salaries [56], while others suggested that incentives worth 45% or 50% of doctors’ salary had the highest coefficient for rural work preference [54, 74, 76]. Nonetheless, some studies found that salary increases had lower utility compared to other recruitment/retention strategies such as good working environment [37], study assistance and supportive management [75], and support for professional development [58]. Opportunities to do private work were associated with better doctor supply or preference to work in rural areas [32, 45, 50, 90].

In term of retention, good salary was the second highest reason for Filipinos doctors’ willingness to stay in rural areas after completing the rural deployment program [96]. Likewise, a 50% salary increase had the highest utility to influence rural retention among Lao doctors [76]. However, in Thailand, financial incentives were deemed less valuable for retention compared to other factors such as working environment, community and personal factors [72]. Nor did an increase in salary associate with Timorese doctors’ preferences to remain working in rural locations [58].

### Personal and professional support

Over half (57%) of the articles—from Bangladesh, China, India, Indonesia, Nepal, Pakistan, Timor-Leste and Vietnam—investigated personal and professional supports. The three most important personal and professional support strategies were working environment, living conditions and career development opportunities. Working environment included adequate facility infrastructure, equipment, drugs, and technology [34, 40, 58, 60, 75, 85, 91], sufficient number of health workers, availability of supportive mentoring or supervision, as well as availability of, and good relationships with, other health professionals [34, 46, 53, 91, 93]. Better housings [76, 92], electricity, water, and communications [52, 57, 59, 88], transportation [57, 72, 74, 76], schooling facilities [50, 53], employment opportunities for spouses [53] were the key living amenities important for doctors to work in rural locations. Clear career promotion schemes such as guarantee of permanent employment, transfer to more developed areas, or promotion opportunities were preferred to overcome rural doctor shortages [31, 54, 56, 76]. While relationship between gender and rural work preference showed mixed results (i.e., the majority found no difference, some found male prefers rural work, and others found the opposite), studies on actual rural work demonstrated that being male was associated with working in rural locations [27, 28]. Another frequently raised issues deterring doctors from rural practice was the lack of security [40, 50, 72, 73, 79, 88, 92], especially among women and in conflict-afflicted areas [57, 83].

### Health systems

Some (13%) studies covered factors related to health systems issues that did not fit well into the existing WHO strategy categories [97]. These were from Bangladesh, China, Cambodia, India, Nepal, Thailand, Timor-Leste, and Vietnam, and included governance, service delivery, and health financing issues impacting on rural workforce supply [32, 50, 59, 70, 73, 83].

### Definitions of rural (or remote)

Definitions of rural used to describe the outcomes were grouped according to four themes identified inductively: (1) inferred with no clear description; (2) facility-related, if differentiated by health facility factors such as facility resources; (3) non-facility-related, if characterized by demographic structure, environmental characteristics, population characteristics, topography or accessibility, and; (4) combination, if combined according to points (2) and (3) above (Table 5).

**Table 5:** Definitions of ‘rural’ as the actual/preferred work locations of Asia-Pacific LMICs doctors and medical students

Definition of rural	Method			Total (%)	Examples of definition
	Quantitative	Qualitative	Others		
No definition	18	6	4	28 (39.4)	The articles either: (1) had no definition of rural or no description of the place characteristics where the study was done and this was not cross-referenced to an earlier study by the authors [66, 68, 69, 74–76, 78, 81, 92], or; (2) relied on respondents’ own definition of rural [33–35, 37, 39, 40, 43–45, 54, 55, 73, 79, 80, 88, 89, 93, 94, 98]
Facility-related					
Type of health facility	9	1	0	10 (14.1)	Township-village health center [47, 95], county or township hospital [61], community or primary or additional primary health centers, secondary hospital [67], community hospital [31, 49], government rural health unit [63, 64]
Non-facility related					
Population size	2	0	0	2 (2.8)	City/municipality with less than 100,000 population [65], district with < 25,000 population [42]
Non-metropolitan	6	0	0	6 (8.5)	Area outside the country capital and/or large city [26–29, 48, 82]
Administrative unit	3	0	0	3 (4.2)	Any area of county, town or village [36]; rural or farther rural [84]; township or rural county [77]
One of the most rural regions in a country	2	4	4	10 (14.1)	Rural relative to other areas in the country, such as: Kampong Chhnang in Cambodia, Guangxi in China [32]; Bac Giang, Lao Cai and Thai Binh in Vietnam [32, 70]; Chattisgarh and Odisha in India [53, 57, 86, 91], East Nusa Tenggara in Indonesia and Zamboanga in the Philippines [32, 51, 53, 57, 62, 70, 86, 90]
Access and/or topography	2	3	0	5 (7.0)	Limited connection to other areas [56], access for transportation [50, 58], mountainous topography, presence of tribal population [52, 83]
Combination of facility and location related					
Type of facility and other characteristics	2	1	0	3 (4.2)	Working in all district and commune-level facilities located outside the country capital [30, 46], area with low population density and poor health facility [59]
Assigned as areas or facilities of doctor shortages	4	0	0	4 (5.6)	Rural posts refer to positions either in selected health facilities or specified areas experiencing doctor shortage [37, 60, 85, 87]

Many included studies (39%) did not provide a definition of rural. Many (39%) also defined ‘rural’ based on non-facility aspects such as level of socioeconomic deprivation [32, 51, 53, 57, 62, 70, 86, 90, 91], being located outside of metropolitan areas [26–29, 48, 82], population size [42, 65], administrative unit definitions [36, 77, 84], and geographical access [50, 52, 56, 58, 83]. Some (14%) studies defined rural as working in primary care or community-level facilities or smaller hospitals [31, 47, 49, 61, 63, 64, 67, 95].

Definitions of rural varied across different studies from the same country. For example, in India, a study conducted in Odisha state considered the entire state as rural [91], while another study conducted in Andhra Pradesh [41], only classified positions in community health centers or lower-level facilities as rural. Likewise, in Indonesia, while one study defined rural districts as < 25,000 population size [42], other studies considered any areas outside of Java and Bali—the most developed regions—as rural, regardless of population size [51, 90]. Studies involving respondents from more than one country relied on respondents’ self-reporting ‘rural’ via questionnaire [33–35].

### Rural preference and career stages

Few studies considered and the impact of career stage. Of 53 studies involving medical graduates, only 2 analyzed outcomes by length of medical career. There was no association between being in early, mid, or later career and intention to stay working rurally among doctors in rural India [91]. Among early career doctors in Thailand, rural preference was higher among a cohort of doctors finishing 2-year compulsory rural service compared to those finishing 1 year [54].

### Study quality

While most of the included studies had a clear research question and coherent methods, some were of poorer quality. Only half (*n* = 26) of 49 quantitative studies applied multivariate analysis (Table 3) with the remainder analyzing data at a univariate level or applied descriptive statistics without adjusting for potential confounding variables. Furthermore, among studies which adjusted for confounders, several relied on subjective definitions of rural location [33–35, 39, 75], thereby reducing study quality. Almost all qualitative studies explained data collection methods and respondent recruitment in detail. However, less than half (*n* = 7) clearly described the theoretical framework used. Some qualitative studies also did not report the relationship between interviewers and respondents, qualifications of the interviewers nor whether training was conducted to ensure consistency in the quality of the interviewing, thereby weakening the credibility of reported findings [99].

## Discussion

This review is the first published study to undertake a detailed synthesis of factors associated with rural medical workforce supply in Asia-Pacific LMICs. Seventy-one articles from 12 countries published between 1999 and 2019 were included. Most evidence was from India, published within the last 10 years and mainly focused on doctors in practice. Around one-third of evidence related to medical students. The spread of evidence was reasonably even across the globally recognized WHO categories of strategies for rural retention, although this review identified a new category: health systems, including government policies and political climate, found to affect the rural medical workforce.

A broad range of educational factors were associated with rural work, especially related to rural background. Both preference and actual work in rural locations were associated with having resided in rural areas during the school-age period, having graduated from a rurally located high school, or being a native of a particular area, consistent with evidence on the importance of recruiting rural-origin students to increase rural doctor supply from other regions [10, 14, 100]. Despite this widely acknowledged evidence, there remains great opportunity for selecting rural background students into medical schools in the Asia-Pacific LMICs. In some countries, such as Indonesia, where more than 50% of medical students are enrolled in private institutions [101] and most medical schools are city-based, executing such rural-focused student selection could require government to provide more financial support for rural students as rural students are less able to afford a medical education. In Thailand, only 1 out of 19 medical schools is privately owned, an easier context in which to implement rural background selection targets [102].

There was limited research isolating the effectiveness of delivering a rural-oriented curriculum, and few examples of rurally located medical schools, rural clerkships, and rural internships. The scant available evidence about rural clerkships showed a positive association with rural work preference; yet, this only applied to those with an urban background. This evidence is not currently strong enough to recommend rural clerkships, nor how to go about rural-focused education. Nonetheless, the evidence available in Asia-Pacific LMICs does suggest that combining rurally based medical education strategies with other strategies, or with other compulsory and incentivizing strategies, can improve rural supply. This is consistent with evidence from other regions that combinations of rural workforce strategies are more effective than single strategies in increasing rural doctor availability [23, 103, 104].

Financial incentives and opportunities to earn income from additional jobs, a conducive work environment, and ongoing supports for professional development were also associated with rural intention, preference, and practice. These benefits were generally coveted by doctors and compensated for the perceived disadvantages of practicing in rural areas. The availability of local amenities such as housing, road infrastructure, and schooling facilities, as well as working-environment considerations such as facility readiness, and adequacy of drugs and equipment, were also associated with doctors’ decisions about work location, supporting the widely documented evidence from around the globe [105]. These strategies—financial incentives, supportive working environments, decent local amenities, and clear career ladder as well as effective human resource management practices—are in the governments’ scope of authority and make practical sense for local governments, rural health services and communities to implement. Thus, this could be especially important because many Asia-Pacific LMICs are decentralized nations in which the management of human resources, health resources, infrastructure, and finance is devolved to local government, thereby providing local governments with a specific role in rural medical workforce management.

While good salary was of high importance for rural doctor retention in the Philippines and Lao [76, 96], studies in Thailand and Timor-Leste found no such association [58, 72]. The majority of respondents in the Thai and Timorese studies had received scholarships (88% and 93%), which may have influenced their preference regarding financial incentives. This calls for more research to explore the role of financial factors, whether given upfront as a scholarship or at the time of employment, in increasing rural doctor retention in Asia-Pacific LMICs.

Past studies indicate that initial employment experiences could play a critical role in influencing doctors’ work performance and retention [18, 106]. In South Africa, doctors who had worked in rural locations at early career stages, even as part of a compulsory assignment, were more likely to have rural work intentions [107]. However, this review identified the paucity of evidence on the association between the different length of employment and actual rural work. A better understanding on the difference of rural work across doctor’s career stages would better inform health workforce planning and decision-making, hence calls for more inquiries on this topic.

We identified that male students or doctors were more likely to prefer or actually work in rural or remote locations [27, 29, 38, 43, 47, 77] and female doctors were more affected by perceptions of lack of security than were male doctors [57, 83]. Since women dominate the doctor population in many Asia-Pacific LMICs [38, 48, 58, 76, 108], understanding the environmental and personal attributes that influence female doctors’ willingness to work rurally is crucial to inform effective policy.

The disparity between the factors related to the two outcomes—preference or actual work in rural areas—was mostly due to difference on variables studied across both outcomes. While there is some evidence that graduation from a public medical school or having lower-income parents was associated with rural work preference, the absence of evidence of these associations for actual work should encourage further investigations. This could become an invaluable policy input, especially for countries where a significant proportion of doctors attended high-fee private schools.

The review identifies some weaknesses of study methodologies. First, almost one-third of the studies did not define rural location, and, for studies that did, the definitions were non-standardized and varied significantly. Some of the definitions used may not reflect geographical and demographic aspects of rurality. This variation may not affect the utility of the study for adding to the emerging evidence about effective rural workforce strategies in Asia-Pacific LMICs; however, it does reduce the capacity to validate and generalize the findings to other contexts. The diverse rural definitions for health policy and research purposes have been previously identified and widely discussed, including in developed countries [24, 109]. The usefulness of future studies in this field could be increased by standardizing definitions of rural.

Second, the majority of included studies only asked whether respondents ever lived in rural areas, without considering any particular rural area. This did not allow any conclusions about whether exposure to any rural area, or familiarity with a specific area, has a stronger influence on doctors’ decisions about where to work. Exploring the nature of doctors’ backgrounds, and whether it is rurality in general, or attachment to a hometown, is an important topic that could provide substantial evidence for policymakers in selecting characteristics for medical students recruitment or doctor deployment.

Finally, the included studies lacked multivariate analyses that is needed to isolate the strength of association of factors related to doctors’ rural work and preference. Adjusting for potential confounders in studies on rural workforce strategies will allow policymakers to understand which of the strategies or sociodemographic characteristics need more emphasis to improve rural doctor availability.

We acknowledge that Asia-Pacific LMICs included in this review range from the world’s most populous countries with strong economic growth (e.g., China) to comparatively smaller, poorer nations with less than 2 million people (e.g., Timor-Leste). Also, no study from Pacific island nations was identified; there remains a need for more studies from these countries. The findings should be generalized with caution given the range of included material from different settings and contexts. By focusing this work on one region, and the context of LMICs being more similar than including all country types, the work substantially adds to the existing evidence for guiding rural medical workforce development in this region, including according to the WHO guidelines.

Almost all of the studies we included involved local-origin doctors, except for Timor-Leste where some doctors are from Cuba. As the poaching of medical staff between high-, middle-, and low-income countries could impact a country’s doctor supply, further research should consider supply chains, including cross-country migration and its impact on different LMICs.

## Conclusions

This study provides critical new evidence, drawn from 20 years’ research, about a range of factors which can be used to target strategies to increase rural medical workforce supply in Asia-Pacific LMICs. The evidence has grown substantially, especially over the last 10 years, but remains confined to 12 Asia-Pacific LMICs. Achieving rural medical workforce growth in Asia-Pacific LMICs required multi-level approaches including selecting more medical students with a rural background, combining this with rural-focused or -located education and return-of-service scholarships, workplace and rural living support and ensuring an appropriately financed rural health system. The review identifies the need for more studies in a broader range of Asia-Pacific countries which define rural clearly, expanding on all strategy areas, use multivariate analyses, and test how various strategies relate to doctor’s career stages.

## Supplementary information

**Additional file 1.** Lists of included article.

**Additional file 2.** Detailed search term for each database searching (June 1990–July 2019).

## Data Availability

All data can be requested from the corresponding author.

## Abbreviations

DCE: Discrete Choice Experiment
LMIC: Low- and middle-income country
LIC: Low-income country
MIC: Middle-income country
PRISMA-Scr: Preferred Reporting Items for Systematic Reviews and Meta-analysis Extension for Scoping Review
WHO: World Health Organization
