# Supplementary material for: Factors associated with increasing rural doctor supply in Asia-Pacific LMICs: a scoping review
Source: Hum Resour Health. 2020 Dec 1;18:93. doi: 10.1186/s12960-020-00533-4 (PMC7706290; doi:10.1186/s12960-020-00533-4)
Supplement: Supplementary file 1 — Additional file 1. Lists of included article. [file 12960_2020_533_MOESM1_ESM.docx]

**Additional File 1. List of included articles**

| **Author** | **Year** | **Title** | **Country** |
| --- | --- | --- | --- |
| R. Gruen; R. Anwar; T. Begum; J. R. Killingsworth; C. Normand | 2002 | Dual job holding practitioners in Bangladesh: an exploration | Bangladesh |
| L. Wang | 2002 | A comparison of metropolitan and rural medical schools in China: which schools provide rural physicians? | China |
| S. Wibulpolprasert; P. Pengpaibon | 2003 | Integrated strategies to tackle the inequitable distribution of doctors in Thailand: four decades of experience | Thailand |
| U. Farooq; A. Ghaffar; I. A. Narru; D. Khan; R. Irshad | 2004 | Doctors perception about staying in or leaving rural health facilities in District Abbottabad | Pakistan |
| K. Butterworth; B. Hayes; B. Neupane | 2008 | Retention of general practitioners in rural Nepal: a qualitative study | Nepal |
| S. Raha; P. Berman; A. Bhatnagar | 2009 | Career preferences of medical and nursing students in Uttar Pradesh | India |
| K. D. Rao; S. Ramani; S. Murthy; I. Hazarika; N. Khandpur; M. Chokshi; S. Khanna; M. Vujicic; P. Berman; M. Ryan | 2010 | Health worker attitudes toward rural service in India: results from qualitative research | India |
| P. R. Shankar | 2010 | Attracting and retaining doctors in rural Nepal | Nepal |
| V. Wiwanitkit; V. Wiwanitkit | 2011 | Mandatory rural service for health care workers in Thailand | Thailand |
| M. Vujicic; B. Shengelia; M. Alfano; H. B. Thu | 2011 | Physician shortages in rural Vietnam: using a labor market approach to inform policy | Vietnam |
| S. Witter; B. Thi Thu Ha; B. Shengalia; M. Vujicic | 2011 | Understanding the 'four directions of travel': qualitative research into the factors affecting recruitment and retention of doctors in rural Vietnam | Vietnam |
| K. Sheikh; B. Rajkumari; K. Jain; K. Rao; P. Patanwar; G. Gupta; K. R. Antony; T. Sundararaman | 2012 | Location and vocation: why some government doctors stay on in rural Chhattisgarh, India | India |
| R. K. Sinha | 2012 | Perception of young doctors towards service to rural population in Bihar | India |
| N. K. Saini; R. Sharma; R. Roy; R. Verma | 2012 | What impedes working in rural areas? A study of aspiring doctors in the National Capital Region, India | India |
| W. Jaskiewicz; O. Phathammavong; P. Vangkonevilay; C. Paphassarang; I. T. Phachanh; L. Wurts | 2012 | Toward development of a rural retention strategy in Lao People’s Democratic Republic: understanding health worker preferences | Lao |
| I. Huntington; S. Shrestha; N. G. Reich; A. Hagopian | 2012 | Career intentions of medical students in the setting of Nepal's rapidly expanding private medical education system | Nepal |
| M. Zimmerman; R. Shakya; B. M. Pokhrel; N. Eyal; B. P. Rijal; R. N. Shrestha; A. Sayami | 2012 | Medical students' characteristics as predictors of career practice location: retrospective cohort study tracking graduates of Nepal's first medical college | Nepal |
| P. R. Shankar; T. P. Thapa | 2012 | Student perception about working in rural Nepal after graduation: a study among first-and second-year medical students | Nepal |
| J. A. Leonardia; H. Prytherch; K. Ronquillo; R. G. Nodora; A. Ruppel | 2012 | Assessment of factors influencing retention in the Philippine National Rural Physician Deployment Program | Philippines |
| F. Cristobal; P. Worley | 2012 | Can medical education in poor rural areas be cost-effective and sustainable: the case of the Ateneo de Zamboanga University School of Medicine | Philippines |
| J. Huang; L. Shi; Y. Chen | 2013 | Staff retention after the privatization of township-village health centers: a case study from the Haimen City of East China | China |
| Z. C. Shroff; S. Murthy; K. D. Rao | 2013 | Attracting doctors to rural areas: a case study of the post-graduate seat reservation scheme in andhra pradesh | India |
| S. Ramani; K. D. Rao; M. Ryan; M. Vujicic; P. Berman | 2013 | For more than love or money: attitudes of student and in-service health workers towards rural service in India | India |
| V. Diwan; C. Minj; N. Chhari; A. De Costa | 2013 | Indian medical students in public and private sector medical schools: are motivations and career aspirations different?–studies from Madhya Pradesh, India | India |
| K. D. Rao; M. Ryan; Z. Shroff; M. Vujicic; S. Ramani; P. Berman | 2013 | Rural clinician scarcity and job preferences of doctors and nurses in India: a discrete choice experiment | India |
| A. Meliala; K. Hort; L. Trisnantoro | 2013 | Addressing the unequal geographic distribution of specialist doctors in indonesia: the role of the private sector and effectiveness of current regulations | Indonesia |
| B. W. Hayes; R. Shakya | 2013 | Career choices and what influences Nepali medical students and young doctors: a cross-sectional study | Nepal |
| B. P. Sapkota; A. Amatya | 2013 | Intended location of future career practice among graduating medical students: perspective from social cognitive career theory in Nepal | Nepal |
| N. Thammatacharee; R. Suphanchaimat; T. Wisaijohn; S. Limwattananon; W. Putthasri | 2013 | Attitudes toward working in rural areas of Thai medical, dental and pharmacy new graduates in 2012: a cross-sectional survey | Thailand |
| M. Lagarde; N. Pagaiya; V. Tangcharoensathian; D. Blaauw | 2013 | One size does not fit all: Investigating doctors' stated preference heterogeneity for job incentives to inform policy in Thailand | Thailand |
| W. Putthasri; R. Suphanchaimat; T. Topothai; T. Wisaijohn; N. Thammatacharee; V. Tangcharoensathien | 2013 | Thailand special recruitment track of medical students: a series of annual cross-sectional surveys on the new graduates between 2010 and 2012 | Thailand |
| D. M. Silvestri; M. Blevins; A. R. Afzal; B. Andrews; M. Derbew; S. Kaur; M. Mipando; C. A. Mkony; P. M. Mwachaka; N. Ranjiti; S. Vermund | 2014 | Medical and nursing students’ intentions to work abroad or in rural areas: A cross-sectional survey in Asia and Africa | Bangladesh, India, Nepal |
| R. A. Dutt; S. Shivalli; M. B. Bhat; J. R. Padubidri | 2014 | Attitudes and perceptions toward rural health care service among medical students | India |
| K. R. Thapa; B. K. Shrestha; M. D. Bhattarai | 2014 | Study of working experience in remote rural areas after medical graduation | Nepal |
| A. D. Asante; N. Martins; M. E. Otim; J. Dewdney | 2014 | Retaining doctors in rural Timor-Leste: a critical appraisal of the opportunities and challenges | Timor Leste |
| E. K. Darkwa; M. S. Newman; M. Kawkab; M. E. Chowdhury | 2015 | A qualitative study of factors influencing retention of doctors and nurses at rural healthcare facilities in Bangladesh | Bangladesh |
| Y. Qing; G. Hu; Q. Chen; H. Peng; K. Li; J. Wei; Y. Yi | 2015 | Factors that influence the choice to work in rural township health centers among 4,669 clinical medical students from five medical universities in Guangxi, China | China |
| S. Nallala; S. Swain; S. Das; S. K. Kasam; S. Pati | 2015 | Why medical students do not like to join rural health service? An exploratory study in India | India |
| I. Syahmar; I. Putera; Y. Istatik; M. A. Furqon; A. Findyartini | 2015 | Indonesian medical students' preferences associated with the intention toward rural practice | Indonesia |
| B. P. Sapkota; A. Amatya | 2015 | What factors influence the choice of urban or rural location for future practice of Nepalese medical students? A cross-sectional descriptive study | Nepal |
| N. Pagaiya; L. Kongkam; S. Sriratana | 2015 | Rural retention of doctors graduating from the rural medical education project to increase rural doctors in Thailand: a cohort study | Thailand |
| W. L. Chuenkongkaew; H. Negandhi; P. Lumbiganon; W. Wang; K. Mahmud; P. V. Cuong | 2016 | Attitude towards working in rural area and self-assessment of competencies in last year medical students: A survey of five countries in Asia | Bangladesh, China, India, Thailand, Vietnam |
| J. Hou; M. Xu; J. C. Kolars; Z. Dong; W. Wang; A. Huang; Y. Ke | 2016 | Career preferences of graduating medical students in China: a nationwide cross-sectional study | China |
| S. Kadam; S. Nallala; S. Zodpey; S. Pati; M. A. Hussain; A. S. Chauhan; S. Das; T. Martineau | 2016 | A study of organizational versus individual needs related to recruitment, deployment and promotion of doctors working in the government health system in Odisha state, India | India |
| K. Sheikh; S. Mondal; P. Patanwar; B. Rajkumari; T. Sundararaman | 2016 | What rural doctors want: a qualitative study in Chhattisgarh state | India |
| F. Efendi; C.-M. Chen; N. Nursalam; N. W. F. Andriyani; A. Kurniati; S. A. Nancarrow | 2016 | How to attract health students to remote areas in Indonesia: a discrete choice experiment | Indonesia |
| E. Keuffell; W. Jaskiewicz; K. Theppanya; K. Tulenko | 2016 | Cost-Effectiveness of Rural Incentive Packages for Graduating Medical Students in Lao PDR | Lao |
| S. A. Rana; M. Sarfraz; I. Kamran; H. Jadoon | 2016 | Preferences Of Doctors For Working In Rural Islamabad Capital Territory, Pakistan: A Qualitative Study | Pakistan |
| M.-F. Smitz; S. Witter; C. Lemiere; P. H.-V. Eozenou; T. Lievens; R. U. Zaman; K. Engelhardt; X. Hou | 2016 | Understanding Health Workers' Job Preferences to Improve Rural Retention in Timor-Leste: Findings from a Discrete Choice Experiment | Timor Leste |
| X. Hou; S. Witter; R. U. Zaman; K. Engelhardt; F. Hafidz; F. Julia; C. Lemiere; E. B. Sullivan; E. Saldanha; T. Palu; T. Lievens | 2016 | What do health workers in Timor-Leste want, know and do? Findings from a national health labour market survey | Timor Leste |
| D. M. Silvestri; M. Blevins; K. A. Wallston; A. R. Afzal; N. Alam; B. Andrews; M. Derbew; S. Kaur; M. Mipando; C. A. Mkony; P. M. Mwachaka; N. Ranjit; S. H. Vermund | 2017 | Nonacademic Attributes Predict Medical and Nursing Student Intentions to Emigrate or to Work Rurally: An Eight-Country Survey in Asia and Africa | Bangladesh, India, Nepal |
| P. P. Reddy; M. S. Anjum; M. Monica; K. Y. Rao; I. A. Hameed; J. R. Reddy | 2017 | Compulsory 1 year rural Service-Stance of interns and postgraduates of medicine and dentistry in Hyderabad City, Telangana: A cross-sectional survey | India |
| R. Vyas; A. Zachariah; I. Swamidasan; P. Doris; I. Harris | 2017 | Evaluation of a distance learning academic support program for medical graduates during rural hospital service in India | India |
| M. R. Behera; C. Prutipinyo; N. Sirichotiratana; C. Viwatwongkasem | 2017 | Interventions for improved retention of skilled health workers in rural and remote areas | India |
| P. R. Rajbangshi; D. Nambiar; N. Choudhury; K. D. Rao | 2017 | Rural recruitment and retention of health workers across cadres and types of contract in north-east India: A qualitative study | India |
| S. B. Halili, Jr.; F. Cristobal; T. Woolley; S. J. Ross; C. Reeve; A. J. Neusy | 2017 | Addressing health workforce inequities in the Mindanao regions of the Philippines: Tracer study of graduates from a socially-accountable, community-engaged medical school and graduates from a conventional medical school | Philippines |
| J. L. Siega-Sur; T. Woolley; S. J. Ross; C. Reeve; A. J. Neusy | 2017 | The impact of socially-accountable, community-engaged medical education on graduates in the Central Philippines: Implications for the global rural medical workforce | Philippines |
| R. Seangrung; P. Chuangchum | 2017 | Factors affecting the rural retention of medical graduates in lower northern Thailand | Thailand |
| R. Arora; P. Chamnan; A. Nitiapinyasakul; S. Lertsukprasert | 2017 | Retention of doctors in rural health services in Thailand: impact of a national collaborative approach | Thailand |
| W. Techakehakij; R. Arora | 2017 | Rural retention of new medical graduates from the Collaborative Project to Increase Production of Rural Doctors (CPIRD): a 12-year retrospective study | Thailand |
| T. Joarder; L. B. Rawal; S. M. Ahmed; A. Uddin; T. G. Evans | 2018 | Retaining Doctors in Rural Bangladesh: A Policy Analysis | Bangladesh |
| J. Liu; B. Zhu; Y. Mao | 2018 | Association between rural clinical clerkship and medical students' intentions to choose rural medical work after graduation: A cross-sectional study in western China | China |
| J. Liu; K. Zhang; Y. Mao | 2018 | Attitude towards working in rural areas: a cross-sectional survey of rural-oriented tuition-waived medical students in Shaanxi, China | China |
| S. Liu; S. Li; R. Yang; T. Liu; G. Chen | 2018 | Job preferences for medical students in China: A discrete choice experiment | China |
| M. R. Behera; C. Prutipinyo; N. Sirichotiratana; C. Viwatwongkasem | 2018 | Living conditions, work environment, and intention to stay among doctors working in rural areas of Odisha state, India | India |
| N. E. Handoyo; Y. S. Prabandari; G. R. Rahayu | 2018 | Identifying motivations and personality of rural doctors: A study in Nusa Tenggara Timur, Indonesia | Indonesia |
| H. Dasman; L. Mwanri; A. Martini | 2018 | Indonesian rural medical internship: The impact on health service and the future workforce | Indonesia |
| T. Woolley; F. Cristobal; J. J. Siega-Sur; S. Ross; A.-J. Neusy; S. D. Halili; C. Reeve | 2018 | Positive implications from socially accountable, community-engaged medical education across two Philippines regions | Philippines |
| P. Boonluksiri; H. Tumviriyakul; R. Arora; W. Techakehakij; P. Chamnan; N. Umthong | 2018 | Community-based learning enhances doctor retention | Thailand |
| L. Jing; K. Liu; X. Zhou; L. Wang; Y. Huang; Z. Shu; J. Lou; J. Fan; X. Sun | 2019 | Health-personnel recruitment and retention target policy for health care providers in the rural communities: A retrospective investigation at Pudong New Area of Shanghai in China | China |
| A. Zhu; S. Tang; N. T. H. Thu; L. Supheap; X. Liu | 2019 | Analysis of strategies to attract and retain rural health workers in Cambodia, China, and Vietnam and context influencing their outcomes | China, Cambodia, Vietnam |
| S. Goel; F. Angeli; N. Dhirar; G. Sangwan; K. Thakur; D. Ruwaard | 2019 | Factors affecting medical students' interests in working in rural areas in North India-A qualitative inquiry | India |
| A. Singh | 2019 | Shortage and inequalities in the distribution of specialists across community health centres in Uttar Pradesh, 2002–2012 | India |
