# Supplementary material for: Factors associated with increasing rural doctor supply in Asia-Pacific LMICs: a scoping review
Source: Hum Resour Health. 2020 Dec 1;18:93. doi: 10.1186/s12960-020-00533-4 (PMC7706290; doi:10.1186/s12960-020-00533-4)
Supplement: Supplementary file 2 — Additional file 2. Detailed search term for each database searching (June 1990–July 2019). [file 12960_2020_533_MOESM2_ESM.rtf]

Additional file 2.
Detailed search term for each database searching (June 1990 – July 2019)

Medline / Pubmed (via Ovid)
1a	In title and abstract:	physician* or doctor* or general practitioner* or medical graduate* or medical practitioner* or medical staff or medical intern* or medical officer* or housemanship or cadetship or clinician* or medical student* or undergraduate medic*	
1b	Subject terms:	Exp Physicians/ or Education, Medical, Graduate/ or "Internship and Residency"/ or Medical Staff/ or exp Medical Staff, Hospital/ or Health Personnel/ or Personnel, Hospital/ or Health Workforce/ or Clinical Clerkship/ or Preceptorship/ or Workforce/ or Students, Medical/	
2a	In title and abstract:	recruit* OR retain* OR retention OR attrition OR attract* OR turnover OR career* OR geographic distribution* OR geographic imbalance* OR work location* OR practice location* OR shortage* OR prefer* OR inten*	
2b	Subject terms:	Choice Behavior/ OR Career Choice/ OR Personnel Management/ or Personnel Turnover/ or Personnel Selection/ or Personnel Administration, Hospital/ or Physician Incentive Plans/ or Job Satisfaction/ or Motivation/ or intention/	
3a	In title and abstract:	rural OR remote OR underserved OR underserviced OR non-metropolitan OR nonmetropolitan OR non-urban OR nonurban OR village OR regional OR tribal	
3b	Subject terms:	Rural Health Services/ or Rural Health/ or Rural Population/ or Professional Practice Location/ OR Medically Underserved Area/	
4a	In all field:	Afghanistan or Bangladesh or Bhutan or Cambodia or China or Chinese or Fiji or India or India* or Indonesia* or Kiribati or Korea or Lao or Malaysia or Maldives or Marshall Island* or Micronesia or Mongolia or Myanmar or Nauru or Nepal* or Pakistan or Palau or Papua New Guinea or PNG or Philippin* or Samoa* or Solomon Island* or Srilanka OR Sri Lanka or Thailand or Thai or Timor Leste or East Timor or Tonga or Tuvalu or Vanuatu or Viet Nam or Vietnam or Asia* or Pacific or Pacific island nations	
4b	In all field:	developing or less-developed OR under-developed OR underdeveloped OR mid* income OR low* income OR deprived OR poor* OR low and mid* income OR lower-mid* income OR upper-mid* income	
4c	Subject terms:	Developing Countries/ OR exp asia/ or exp oceania/ 
+
Pacific islands/	
	Final search	(1a OR 1b) AND (2a OR 2b) AND (3a OR 3c) AND (4a OR 4b OR 4c)	


EMBASE
1a	In title and abstract:	physician* or doctor* or general practitioner* or medical graduate* or medical practitioner* or medical staff or medical intern* or medical officer* or housemanship or cadetship or clinician* or medical student* or undergraduate medic*	
1b	Emtree	'physician'/exp OR 'medical personnel'/de OR 'medical staff'/de OR 'health workforce'/de OR 'clinician'/de OR 'health care personnel'/de OR 'medical student'/de 	
2a	In title and abstract:	recruit* OR retain* OR retention OR attrition OR attract* OR turnover OR career* OR geographic distribution* OR geographic imbalance* OR work location* OR practice location* OR shortage* OR prefer* OR inten*	
2b	Emtree	'medical education'/de OR 'personnel management'/de OR 'health care personnel management'/de OR ' decision making'/de OR 'job satisfaction'/de OR 'motivation'/de OR 'career'/de OR 'geographic distribution'/de OR 'health care distribution'/de OR 'hospital personnel management'/de OR 'preference'/de	
3a	In title and abstract:	rural OR remote OR underserved OR underserviced OR non-metropolitan OR nonmetropolitan OR non-urban OR nonurban OR village OR regional OR tribal	
3b	Emtree	'rural area'/de OR 'rural health care'/de OR 'rural hospital'/de OR 'rural population'/de	
4a	In all field:	Afghanistan or Bangladesh or Bhutan or Cambodia or China or Chinese or Fiji or India or India* or Indonesia* or Kiribati or Korea or Lao or Malaysia or Maldives or Marshall Island* or Micronesia or Mongolia or Myanmar or Nauru or Nepal* or Pakistan or Palau or Papua New Guinea or PNG or Philippin* or Samoa* or Solomon Island* or Srilanka OR Sri Lanka or Thailand or Thai or Timor Leste or East Timor or Tonga or Tuvalu or Vanuatu or Viet Nam or Vietnam or Asia* or Pacific or Pacific island nations	
4b	In all field:	developing or less-developed OR under-developed OR underdeveloped OR mid* income OR low* income OR deprived OR poor* OR low and mid* income OR lower-mid* income OR upper-mid* income	
4c	Emtree	'asia pacific'/de OR 'asia pacific region'/de OR 'low middle income country'/de OR 'developing country'/de OR 'South Asia'/exp OR 'south east asia'/de OR 'Pacific islands'/exp OR 'East Asian'/exp OR 'Southeast Asia'/exp OR 'western pacific'/de OR 'western pacific region'/de	
	Final search	(1a OR 1b) AND (2a OR 2b) AND (3a OR 3c) AND (4a OR 4b OR 4c)	


CINAHL
1a	In title and abstract:	physician* or doctor* or general practitioner* or medical graduate* or medical practitioner* or medical staff or medical intern* or medical officer* or housemanship or cadetship or clinician* or medical student* or undergraduate medic*	
1c	Subject terms:	(MH "Physicians+") OR (MH "Medical Staff+") OR (MH "Interns and Residents") OR (MH "Internship and Residency") OR (MH "Students, Medical") 	
2a	In title and abstract:	recruit* OR retain* OR retention OR attrition OR attract* OR turnover OR career* OR geographic distribution* OR geographic imbalance* OR work location* OR practice location* OR shortage* OR prefer* OR inten*	
2b	Subject terms:	(MH "Career Planning and Development") OR (MM "Education, Medical") OR (MH “Job Satisfaction”) OR (MH “Personnel Turnover”) OR (MH "Personnel Recruitment") OR (MH "Personnel Selection") OR (MH "Physician Incentive Plans") OR (MH “Motivation”)	
3a	In title and abstract:	rural OR remote OR underserved OR underserviced OR non-metropolitan OR nonmetropolitan OR non-urban OR nonurban OR village OR regional OR tribal	
3b	Subject terms:	(MH "Rural Health Services") OR (MH "Medically Underserved Area") OR (MH "Hospitals, Rural") OR (MH "Geographic Locations") OR (MH "Rural Health Centers") OR (MH "Rural Health Personnel")	
4a	In all text	TX Afghanistan or Bangladesh or Bhutan or Cambodia or China or Chinese or Fiji or India or India* or Indonesia* or Kiribati or Korea or Lao or Malaysia or Maldives or Marshall Island* or Micronesia or Mongolia or Myanmar or Nauru or Nepal* or Pakistan or Palau or Papua New Guinea or PNG or Philippin* or Samoa* or Solomon Island* or Srilanka OR Sri Lanka or Thailand or Thai or Timor Leste or East Timor or Tonga or Tuvalu or Vanuatu or Viet Nam or Vietnam or Asia* or Pacific or Pacific island nations	
4b	In all text	developing or less-developed OR under-developed OR underdeveloped OR mid* income OR low* income OR deprived OR poor* OR low and mid* income OR lower-mid* income OR upper-mid* income	
4c	Subject terms:	(MH "Sri Lanka") OR (MH "Pakistan") OR (MH "Nepal") OR (MH "India") OR (MH "Bhutan") OR (MH "Bangladesh") OR (MH "Asia, Southeastern+") OR (MH "Afghanistan") OR (MH "China+") OR (MH "Mongolia") OR (MH "North Korea") OR (MH "Papua New Guinea") OR (MH "Pacific Islands") OR (MH "Independent State of Samoa") OR (MH "Samoa") 	
4d	Subject terms:	(MH "Developing Countries") OR (MH "Low and Middle Income Countries")	
	Final search	[(1a AND 1b) OR (1c)] AND (2a OR 2b) AND (3a OR 3b) AND (4a OR 4b OR 4c OR 4d)	

PsycINFO (via Ovid)
1a	In title and abstract:	physician* or doctor* or general practitioner* or medical graduate* or medical practitioner* or medical staff or medical intern* or medical officer* or housemanship or cadetship or clinician	
1b	Subject terms:	Physicians/ or General Practitioners/ or Physicians, Family/ or Physicians, Primary Care/ or Education, Medical, Graduate/ or "Internship and Residency"/ or Medical Staff/ or exp Medical Staff, Hospital/ or Health Personnel/ or Personnel, Hospital/ or Health Workforce/ or Clinical Clerkship/ or Preceptorship/ 	
2a	In title and abstract:	recruit* OR retain* OR retention OR attrition OR attract* OR turnover OR career* OR geographic distribution* OR geographic imbalance* OR work location OR practice location OR shortage* Choice Behavior/ OR Career Choice/ OR  Personnel Management/ or Personnel Turnover/ or Personnel Selection/ or Personnel Administration, Hospital/ or Physician Incentive Plans/ or Job Satisfaction/ or Motivation/	
2a	In title and abstract:	rural OR remote OR underserved OR underserviced OR non-metropolitan OR nonmetropolitan OR non-urban OR nonurban OR village OR regional OR disadvantaged	
2b	Subject heading:	Rural Health Services/ or Rural Health/ or Rural Population/ or Professional Practice Location/ OR Medically Underserved Area/	
3a	In title and abstract:	physician* or doctor* or general practitioner* or medical graduate* or medical practitioner* or medical staff or medical intern* or medical officer* or housemanship or cadetship	
3b	Subject heading:	Physicians/ or General Practitioners/ or Physicians, Family/ or Physicians, Primary Care/ or Education, Medical, Graduate/ or "Internship and Residency"/ or Medical Staff/ or exp Medical Staff, Hospital/ or Health Personnel/ or Personnel, Hospital/ or Health Workforce/ or Clinical Clerkship/ or Preceptorship/ 	
4a	In all field:	Afghanistan or Bangladesh or Bhutan or Cambodia or China or Chinese or Fiji or India or India* or Indonesia* or Kiribati or Korea or Lao or Malaysia or Maldives or Marshall Island* or Micronesia or Mongolia or Myanmar or Nauru or Nepal* or Pakistan or Palau or Papua New Guinea or PNG or Philippin* or Samoa* or Solomon Island* or Srilanka OR Sri Lanka or Thailand or Thai or Timor Leste or East Timor or Tonga or Tuvalu or Vanuatu or Viet Nam or Vietnam or Asia* or Pacific or Pacific island nations	
4b	In all field:	developing or less-developed OR under-developed OR underdeveloped OR mid* income OR low* income OR deprived OR poor* OR low and mid* income OR lower-mid* income OR upper-mid* income	
4c	Subject heading:	'asia pacific'/de OR 'asia pacific region'/de OR 'low middle income country'/de OR 'developing country'/de OR 'South Asia'/exp OR 'south east asia'/de OR 'Pacific islands'/exp OR 'East Asian'/exp OR 'Southeast Asia'/exp OR 'western pacific'/de OR 'western pacific region'/de	
	Final search	(1a OR 1b) AND (2a OR 2b) AND (3a OR 3c) AND (4a OR 4b OR 4c)	

Scopus, HRH Journal, Rural and Remote Health Journal, Google Scholar
1		Physician OR doctor OR “general practitioner” OR “medic* graduate OR “junior doctor” OR “junior physician” OR “early career doctor” OR “early career physician” OR “young doctor” OR “young physician”	
2		Recruitment OR retention OR factor OR determinant OR predictor OR turnover OR “career choice*” OR “geographic distribution”	
3		Rural OR remote OR underserved	
	Final search	1 AND 2 AND 3	
